# Reconstruction of central aortic pressure based on TCN-attention model

**DOI:** 10.3389/fphys.2025.1693431

**Published:** 2025-10-16

**Authors:** Wenyan Liu, Yajie Cao, Yali Fu, Shuo Du, Chuanchao Wu, Ye Tian, Liyuan Zhang, Yi Liu, Lisheng Xu, Zhiguo Gui

**Affiliations:** ^1^ School of Information and Communication Engineering, North University of China, Taiyuan, China; ^2^ College of Electronic Information Engineering, Hebei University, Baoding, China; ^3^ College of Information Science and Engineering, Northeastern University, Shenyang, China

**Keywords:** deep learning, central aortic pressure, radial arterial pressure, temporalconvolution network, attention mechanism

## Abstract

**Introduction:**

Among the causes of cardiovascular diseases, abnormal blood pressure is especially significant. Blood pressure is a crucial hemodynamic biomarker of the cardiovascular health. Central aortic blood pressure correlates more closely with cardiovascular disease than peripheral arterial blood pressure. It can reflect the status of coronary arteries and aortas more directly and accurately, making it a significant tool for assessing cardiovascular risks. Invasive central aortic blood pressure measurement is considered the “gold standard” for evaluating left ventricular and coronary artery loads. However, due to the invasive and high cost of consumables, the widespread use of central aortic blood pressure is hindered in primary medical institutions and among large populations. Traditional non-invasive methods also have some limitations.

**Methods:**

This paper proposes reconstructing central aortic pressure based on the TCN-Attention model, which primarily extracts local patterns from the time series. Simultaneously, the attention mechanism focuses on extracting global patterns to compensate for the shortcomings of the TCN model, which cannot perform global feature extraction. It efficiently extracts local patterns in the time series data that characterize mutations and other key time points and global patterns that indicate trends and periodicity, thus enabling the efficient reconstruction of central aortic pressure.

**Results:**

The experimental results demonstrate that the improved TCN-Attention model presented in this paper is more accurate than the TCN model.

**Disussion:**

The precise measurement of central aortic pressure has significant clinical value in preventing, diagnosing, and treating cardiovascular diseases.

## 1 Introduction

The latest statistical data of the World Health Organization shows that cardiovascular diseases account for a considerable proportion of total global deaths. There are numerous types of cardiovascular diseases, including coronary heart disease, hypertensive heart disease, arrhythmia, etc (Vos et al., 2020) (World Health Organization, 2018). These diseases not only seriously threaten life and health, but also bring heavy economic and psychological burdens to patients and their families. Concurrently, these conditions impose substantial strain on public healthcare resources. Blood pressure abnormalities are highly critical among the causative factors of cardiovascular diseases. Blood pressure serves as a pivotal physiological parameter for cardiovascular health assessment, simultaneously reflecting cardiac pumping efficiency and the hemodynamic stress exerted on vascular walls. Maintaining normal blood pressure is essential for ensuring adequate blood perfusion and the proper functioning of all organs. When blood pressure remains at abnormal levels for a prolonged period, either hypertension or hypotension, it significantly increases the risk of cardiovascular disease. For example, high blood pressure will increase the load on the heart, leading to myocardial hypertrophy, damaging the endothelial of blood vessels, accelerating the process of atherosclerosis, which may lead to coronary heart disease, stroke, and other serious complications. Concurrently, numerous studies have shown that central aortic pressure (CAP) is superior to peripheral arterial pressure in assessing the effectiveness of anti-hypertensive treatments, predicting cardiovascular events, and guiding clinical decision-making (Roman et al., 2007). CAP is the lateral pressure exerted on the proximal vascular structures of the aorta, which is affected by factors such as left ventricular ejection, arterial resistance, and peripheral arterial load. Compared to peripheral arterial blood pressure, CAP correlates more closely with cardiovascular diseases and provides a more direct and accurate reflection of the state of the coronary artery and the aorta, making it a primary tool for assessing cardiovascular risks (Roman et al., 2007). Additionally, CAP can serve as a reference indicator for evaluating the efficacy of anti-hypertensive medications (McGaughey et al., 2016). Therefore, accurate CAP measurement has significant clinical value in preventing, diagnosing, and treating cardiovascular diseases.

Existing measurement methods include both invasive and non-invasive approaches. Invasive measurement methods use a catheter, which is fed into the radial or femoral artery to reach the proximal aorta. Its terminal is linked to an external pressure sensor. The pressure is delivered from the blood vessel to the external transducer under liquid pressure. The external transducer utilizes analog-to-digital conversion to convert the transducer pressure into digital data for further processing. These data are processed by collection systems or computers that can provide real-time, dynamic blood pressure readings. Therefore, invasive CAP measurements are considered the “gold standard” for evaluating the load on the left ventricle and coronary arteries. However, cardiac catheterization and pressure guide-wire techniques are invasive operations that can injure the blood vessels and the heart. They may lead to complications such as bleeding at the puncture site, vascular tears and infections. In severe cases, arrhythmias, acute myocardial infarction, aortic coarctation, and other life-threatening injuries may occur, such as aortic coarctation. It is primarily used for critically ill patients and those undergoing cardiac surgery. Moreover, the technique of operators must be strictly trained, and experienced and specialized interventionists must be involved to ensure the successful completion of the surgery. Invasive CAP measurements also require expensive X-ray fluoroscope equipment, catheterization laboratories, and other specialized equipment. The cost of consumables is very high, which prevents their widespread adoption in primary medical institutions and among large populations.

Non-invasive measurement methods provide key tools for cardiovascular evaluation through pulse wave analysis, ultrasound, and other techniques (Morgan et al., 2004). Although calibration and individualization difficulties exist, the advantages of non-invasive and dynamic monitoring make it increasingly widely used in clinics and research. Non-invasive measurement techniques utilize the hemodynamic relationship between CAP and peripheral arterial pressure to establish mathematical models, and use peripheral arterial blood pressure to estimate CAP^.^ Nowadays, non-invasive techniques have become the primary method for measuring CAP due to their practical and safe properties. Substituting, transferring functions, and multi-channel blinded system identification methods are commonly used. Peripheral arterial pressure is often used as a substitute for CAP due to its similarity to CAP, much like the use of the carotid artery (Zhou et al., 2024) (Kroeker and Wood, 1955). Although the substitution method has some promise in clinical practice, it can produce results similar to those obtained by direct measurement under certain circumstances. However, the technique has specific errors and limitations; more accurate measurements are needed in the clinic. Therefore, the method can only serve as an approximate estimation and cannot fully replace CAP. For this reason, the transfer function method is introduced with the multi-channel blind system identification method.

A quantifiable mathematical relationship exists between the peripheral arterial pulse wave and the central aortic pulse wave. The central aortic pressure waveform is estimated by modeling the propagation and reflection effects of the waveform through the establishment of a transfer function model (Cameron et al., 1998). It describes how the central aortic pressure waveform is transmitted and converted to the function of the peripheral arterial blood pressure waveform by the action of the heart and vascular system. It establishes a quantitative relationship between CAP and peripheral arterial pressure. The most widely used technique is the generalized transfer function to convert the peripheral arterial blood pressure waveform collected into the theoretical central arterial waveform, thereby indirectly deriving the CAP, which is the core technique for non-invasive CAP measurement (Payne et al., 2007). This technique is efficient and safe, and avoids the risks and complications associated with invasive catheterization. However, this method has limitations because the real arterial system may be nonlinear under high blood flow or pathological conditions, resulting in different arterial branching patterns among individuals and the location of reflection points, thereby influencing the transmission of waveform. Therefore, the results presented by the data are of very low precision. Moreover, the generalized transfer function is based on data from healthy individuals. It does not consider special populations, so the error for these populations, such as those with aortic stenosis and severe atherosclerosis, will be substantial and cannot be fully applied to all people. The accuracy will be limited by the individual and the pathological state. Therefore, this type of method is not widely used in clinical practice. The multi-channel blind system identification method regards the cardiovascular system as a system model with a single input and multiple outputs, by measuring peripheral arterial blood pressure at numerous locations and employing a blind identification algorithm to determine the channel parameters of system. This method eliminates the need to build a model and directly measures peripheral arterial blood pressure to reconstruct CAP. Blind identification refers to the fact that there is no need to know the input signals of the system or the parameters of the *a priori* model in advance. The key to this technique is to use the statistical independence of signal or non-Gaussian properties to isolate the system response or use matrix decomposition to obtain the features of system. These properties bring advantages in resisting noise, displaying hidden features, and dealing with complex systems. However, simultaneous measurements of multiple peripheral signals also significantly inconvenience clinical applications.

This paper proposes a non-invasive central aortic pressure waveform estimation method based on a single-channel radial arterial pressure waveform (Du et al., 2022; Zeiler and Fergus, 2014; Szegedy et al., 2015; Wang et al., 2012). The method builds upon the temporal convolutional network (TCN) model, incorporates an attention mechanism (Wang et al., 2025; Chen et al., 2025), and further optimizes it to propose the TCN-Attention model, thereby enhancing the model’s capability to focus on channel information. The innovation of this method is that it effectively combines the advantages of TCN and the attention mechanism. The TCN model is mainly responsible for extracting the local patterns of the time series. At the same time, the attention mechanism focuses on extracting the global patterns to make up for the shortcomings of the TCN model, which cannot carry out the global feature extraction, thus realizing the efficient reconstruction of the CAP. Moreover, the introduction of the attention mechanism allows the model not only to capture the local dependencies in the sequence data by stacking the convolutional layers and increasing the sensory field of the convolutional kernel, but also allows the neural network to assign different weights to different moments of the input sequence data, so that it pays more attention to historical moments related to the prediction results, thus achieving the balance between the extraction of local features and global features.

## 2 Methods

The main structures of the TCN-Attention model are causal inflationary convolution and residual module. The TCN-Attention model has the properties of a concise form of convolution suitable for sequence modeling, memory for history, and consistent output and input dimensions of the model. The input sequence is denoted as $\left[\;x_{0},\cdots ,x_{N}\right]$ , and the output sequence is predicted as $\left[{\overset{\wedge }{\;y}}_{0},\cdots ,{\overset{\wedge }{y}}_{N}\right]$ . In this way, the sequence model between input and output can be represented as in Equation 1:

$${\overset{\wedge }{y}}_{n}=G\left(x_{0},\cdots ,x_{n}\right),0\leq n\leq N$$ (1)

In Equation 1, *G* denotes the sequence model obtained from the training dataset; ${\overset{\wedge }{y}}_{n}$ denotes the value of the predicted output sequence at the *nth* moment. The model’s parameters can be obtained by minimizing the loss function $L\left(y,\overset{\wedge }{y}\right)$ , where the actual output sequence is $y=\left[y_{0},\cdots ,y_{N}\right]$ and the predicted output sequence is $\overset{\wedge }{y}=\left[{\overset{\wedge }{y}}_{0},\cdots ,{\overset{\wedge }{y}}_{N}\right]$ .

### 2.1 Causal inflation convolution

The input sequence signal is denoted as $x=\left[x_{0},\cdots ,x_{N}\right]$ , and the output value of causally inflated convolution at moment *n,* $F\left(n\right)$ is described as in Equation 2:

$$F\left(n\right)=\sum_{i=0}^{k-1}f\left(i\right)\cdot x\left(n-d\cdot i\right)$$ (2)

Thereof, $d=2^{l}$ is the inflation factor, where 0≤l≤L is the number of network convolution layers, $f:\left\{0,\cdots ,k-1\right\}$ denotes the filter, and k is the convolution kernel size; ( n−d·i ) indicates the direction of past time. To effectively utilize the information from long input sequence signals, the receptive field of the TCN model is increased by increasing the larger filter size and expansion factor. The effective history receptive field of each layer is $\left(k-1\right)\cdot d$ , and the sum of the receptive fields of all convolutional layers is:

$$R=1+\sum_{l=0}^{L-1}\left(k-1\right)\cdot d=1+\sum_{l=0}^{L-1}\left(k-1\right)\cdot 2^{l}$$ (3)

To cover the length of the input sequence signal N , the receptive field R must be greater than or equal to *N*, in Equation 3 and the number of convolutional layers L must be greater than or equal to $\log_{2}\left(\frac{N+k-2}{k-1}\right)$ , which can be obtained by solving Equation 4.

$$\left(1+\sum_{l=0}^{L-1}\left(k-1\right)\cdot d\right)\geq N$$ (4)

**FIGURE 1:**
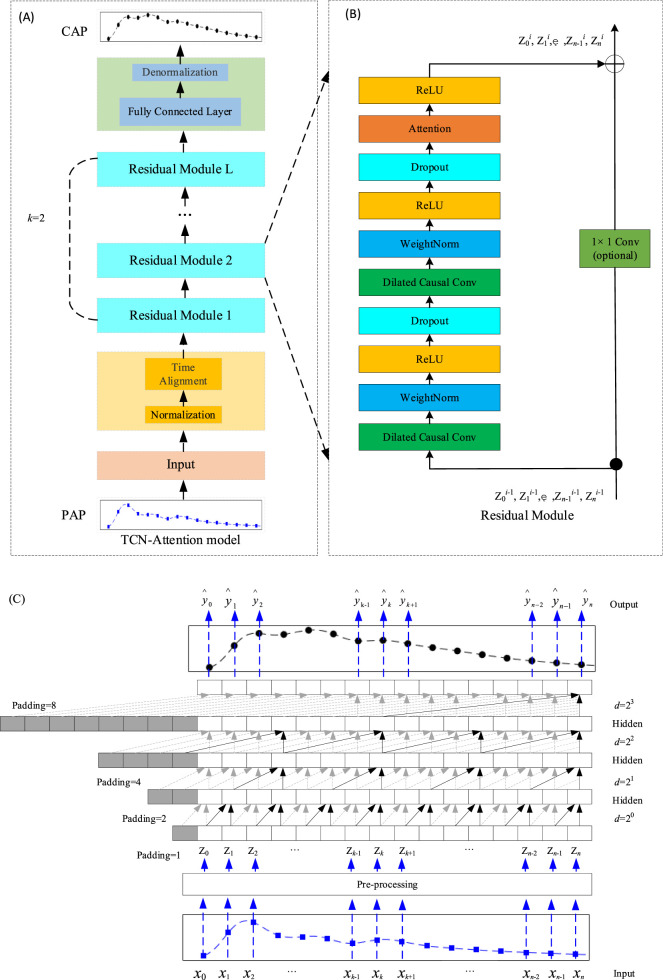
Structure of the TCN-Attention model. **(A)** The overall architecture of the TCN-Attention model, superimposed by the residual module, where the convolution kernel size *k*= 2 and the expansion factor *d*= 2^*l*^ (*l*= 0, 1, …, L-1); **(B)** The residual module of the TCN-Attention model. If the dimensions of the inputs and outputs are different, they need to be manipulated with a 1 × 1 convolution, here Z^*l*^ denotes the output of the *lth* module; **(C)** An example of causal convolution with a hidden layer *L* of 4, a convolution kernel of size *k* of 2 (as indicated by the arrows) and an expansion factor *d* = 1, 2, 4, 8. The sensory field for each layer of the convolution is $\left(k-1\right)\cdot d$ .

As shown in Figure 1A, the TCN-Attention model is an integrated deep learning architecture that combines Temporal Convolutional Network (TCN) with attention mechanisms. As shown in Figure 1C, an example represents the causal inflation convolution. The TCN-Attention model requires that the output and the input have the same length. To ensure that the output tensor has the same length as the input tensor, zero-padding on the left side of the input tensor is required, and causal convolution can be guaranteed. In a convolutional neural network, *p* denotes padding; *s* denotes step size; in this paper, *s* = 1. The size of the sequence $N^{\prime }$ after causal expansion convolution is represented in Equation 5:

$$N^{\prime }=\frac{N+2p-k^{\prime }}{s}+1=\frac{N+2p-\left[k+\left(k-1\right)\cdot \left(d-1\right)\right]}{s}+1$$ (5)

After the convolution operation, the TCN-Attention model will have a “chomp1d” function to cut the excess padding. Therefore, the size of the output after processing by “chomp1d “ N ” is represented as in Equation 6:

$$N^{\prime \prime }=N^{\prime }-p=N+p-\left(k-1\right)\cdot d$$ (6)

Therefore, to ensure that the length of the input and the output are the same, it is required that the size of $N^{\prime \prime }=N$ , the input is $p=\left(k-1\right)\cdot d$ .

### 2.2 Residual module

Features can be enhanced by increasing the number of network layers to obtain more helpful information. However, experiments have found that the optimization effect worsens as the network is deepened, and testing and training accuracy are reduced. This phenomenon occurs because deepening the network causes the gradient explosion or a gradient vanishing. To train deeper networks, Kaiming He proposed a new network structure, ResNet (He et al., 2016), as shown in Figure 1B, each residual block having two branches. The first branch consists of two causally inflated convolutional layers, two weight normalization layers (WeightNorm), two activation function ReLU layers, and two Dropout layers (Gu et al., 2018), where the WeightNorm layer is used to normalize the inputs of the implicit layer to counteract the problem of gradient explosion. The activation function ReLU introduces the nonlinearity. Dropout regularization is added to prevent overfitting (Lin et al., 2021). The attention mechanism is also introduced in the first branch. The second branch is the skip connection of inputs. For the standard ResNet model, the inputs are added directly to the output of the residual function. However, for the TCN-Attention model, the residual block input and output dimensions may not match. Therefore, a 1 × 1 convolution layer is used to ensure that the inputs are subjected to a dimensionality upgrading operation, which allows for the computation of summation between input and output sequences of the same dimensionality (Lin et al., 2021) (Bai et al., 2018). To train the model to optimality, the loss function defined in this chapter is Mean Squared Error (MSE) as in Equation 7:

$$L=\frac{1}{N}\sum_{n=0}^{N-1}{\left(y_{n}-{\overset{\wedge }{y}}_{n}\right)}^{2}$$ (7)

Here $y_{n}$ , ${\overset{\wedge }{y}}_{n}$ respectively, denote the measured and estimated central aortic pressure. To evaluate the accuracy of the proposed TCN-Attention model for reconstructing central aortic pressure waveforms, the Root Mean Squared Error (RMSE) was used here as an index for assessment, and the significance between the estimated and real measured central aortic pressure waveforms was analyzed by a paired t-test (IBM SPSS Statistics, Version-23). The correlation was analyzed by using Spearman’s correlation coefficient. A p-value <0.05 was considered statistically significant.

## 3 Experimental datasets and pre-processing

### 3.1 Datasets

Subjects were recruited from the Northeastern University and a community at Shenyang, China. Approval has been obtained from the Research Ethics Committee of the Northeastern University (EC-2020B017), and all participants gave written informed consent. The inclusion criterion for this study was age between 18 and 50 years. The exclusion criteria included age under 18 years, severe organic diseases of the heart, liver, or kidneys, and mental illness or cognitive impairment. The experiment included 1032 individuals with a mean age of 28.4 ± 16.4, height of 171.0 ± 33.4 cm, weight of 69.6 ± 21.1 kg, and other parameters as shown in Table 1. The data collection process was divided into two main steps: first, the subjects were seated and kept in a stable position, and the systolic and diastolic blood pressure were collected three times from the subjects to be averaged by using a mercury sphygmomanometer; and second, the radial artery pulse wave was collected at least twice. After each acquisition, if the built-in signal quality assessment parameter Operator Index was less than 80, the instrument was reacquired to ensure that the Operator Index of the two data sets was greater than or equal to 80. The radial artery pulse wave was collected with the SphygmoCor CVMS device from AtCor (Australia), which utilized a Millar tonometer (Millar Instruments, Houston, United States) force transducer to capture radial artery pulse waves, with a sampling frequency of 128 Hz (Ding et al., 2011). Using a high-fidelity pressure transducer, the device can record pulse waveform at superficial arteries, most commonly the radial artery, followed by the carotid, brachial, or femoral arteries (Zuo et al., 2010). This peripheral arterial waveform is then converted to an estimated central aortic waveform by applying a generalized conversion function or a patient-specific calibrated mathematical model to derive parameters such as central systolic, diastolic, and pulse pressure.

**TABLE 1:** Clinical characteristics of the subjects.

Parameter	Mean ± standard deviation
Sample size	1,032
Age	28.4 ± 16.4
Height (cm)	171.0 ± 33.4
Weight (kg)	69.6 ± 21.1
Body mass index (kg/m2)	23.1 ± 7.6
Heart rate (bpm)	67.5 ± 9.8
Systolic blood pressure (mmHg)	119.8 ± 15.0
Diastolic blood pressure (mmHg)	75.1 ± 9.8

**FIGURE 2:**
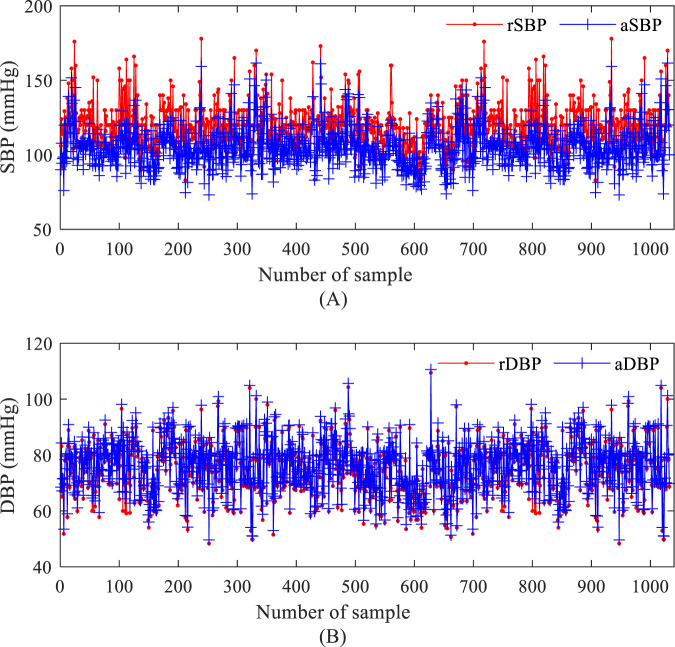
Blood pressure values based on the measurement data set. **(A)** systolic blood pressure of the radial artery and systolic blood pressure of the central artery; **(B)** diastolic blood pressure of the radial artery and diastolic blood pressure of the central artery.

The distribution of systolic (SBP) and diastolic (DBP) values in the measurement dataset (NEU-PWDB) is shown in Figure 2. The systolic blood pressure of the radial artery (SBP of the radial artery, rSBP) was higher than the systolic blood pressure of the central artery (SBP of the central aorta, aSBP), ranging from 0 mmHg to 43 mmHg (Mean ± SD, 14.59 ± 5.95 mmHg). The diastolic blood pressure of the radial artery (DBP of the radial artery, rDBP) and the diastolic blood pressure of the central aorta (DBP of the central aorta, aDBP) were essentially the same. Even in some individuals, the aDBP was slightly higher than the rDBP, with a range of 0.20 mmHg–14 mmHg (Mean ± SD, 1.40 ± 1.02 mmHg). The experimental statistics were also consistent with the “arterial pressure amplification phenomenon,” (Ohte et al., 2007) the mean difference between systolic blood pressure in the central and peripheral arteries was approximately 15 mmHg. The dataset was randomly partitioned into a training set (70%) for model parameter optimization and a testing set (30%) for the final, unbiased evaluation of generalization performance, with the latter kept completely separate and never accessed during model tuning.

### 3.2 Data pre-processing

A notable challenge in studies using pulse waveform to predict blood pressure is that the range of blood pressure values varies widely between individuals, e.g., systolic blood pressure in healthy adults typically fluctuates between 90 and 140 mmHg. By contrast, values in patients with hypertension or hypotension may fall well above or below this range. This wide distribution of values threatens the training stability of deep learning models, potentially causing gradient explosion or gradient vanishing, thus hindering effective model convergence and prediction accuracy. Therefore, normalizing the raw pulse wave data before model training is crucial. One of the core tasks of this process is to normalize the range of values in the pulse wave signal that characterizes the blood pressure amplitude. This normalization is significant not only for making the data format conform to the model input requirements, but also for its ability to improve the training efficiency significantly by compressing the numerical spans, so that the optimization algorithms can find stable and effective solutions faster and accelerate the model to reach the performance plateau. At the same time, normalization is also a key strategy to improve data quality, which helps to suppress all kinds of noise interference (including physiological fluctuations, equipment measurement errors, and individual baseline differences) mixed in the signal, to more clearly extract the practical waveform features reflecting the state of blood pressure. To make the training data format meet the requirements of the model, this paper standardizes the acquired data sequences before model training, normalizes the range of blood pressure amplitude in the pulse waveform sequences, accelerates the training speed of the model, and quickly reaches the optimized stable state. The purpose is to improve the data quality, reduce the noise interference, and extract the practical features.

There are four standard methods for pre-processing time series data:

#### 3.2.1 Min-max normalization

Min-max normalization, or deviation normalization, is a linear transformation of the original data. So that the data features are mapped to [0, 1]. The transformation function is represented as in Equation 8:

$$\tilde{x}=\frac{x-\min\left(x\right)}{\max\left(x\right)-\min\left(x\right)}$$ (8)

#### 3.2.2 Mean-variance normalization

Mean-variance standardization, also known as Z-score standardization, is data standardization through the mean and standard deviation of the original data. After the data are pre-processed to follow a standard normal distribution, i.e., the mean is 0, the standard deviation is 1, the transformation function is represented as in Equation 9:

$$\tilde{x}=\frac{x-\text{mean}\left(x\right)}{\text{std}\left(x\right)}$$ (9)

#### 3.2.3 Mean value standardization

Mean value standardization, the data features are mapped to between [-0.5, 0.5], the transformation function is represented as in Equation 10:

$$\tilde{x}=\frac{x-\text{mean}\left(x\right)}{\max\left(x\right)-\min\left(x\right)}$$ (10)

#### 3.2.4 Unit Norm Scaling

Unit Norm Scaling, which equals the mode of the data features to 1, the transformation function is represented as in Equation 11:

$$\tilde{x}=\frac{x}{\text{norm}\left(x\right)}$$ (11)

In the above four different data pre-processing methods, max indicates the operation of taking the maximum value; min suggests the operation of taking the minimum value; mean indicates the operation of taking the mean value; std suggests the operation of taking the variance; and norm indicates the operation of taking the mode. The study shows that after pre-processing the pulse sequence data using mean-variance normalization, mean normalization, and unit normalization, the pulse waveform predicted by the model shows a peak shaving phenomenon, i.e., the peaks and valleys can not be estimated, and a straight line phenomenon occurs here. This phenomenon is because the mean-variance normalization, mean - value normalization, and unit-normalization pre-processing methods are easily affected by the minimum and maximum values of the pulse waveform. If the data exceeds the range of the extreme values of the training set, it cannot be processed effectively. In this paper, minimum-maximum normalization is chosen as the best pre-processing method. However, the minimum-maximum normalization method also has a drawback; when new data is added, it may lead to changes by calculating the maximum and minimum values, which need redefined. This paper uses minimum-maximum normalization in Equations 12, 13, where the minimum and maximum values are taken from all sample inputs and outputs.

$$\left\{\begin{array}{l} {\tilde{x}}_{i}=\frac{x_{i}-a}{b},\\ {\tilde{y}}_{i}=\frac{y_{i}-a}{b}, \end{array}\right.\begin{array}{cc} & i=0,1,2,... \end{array},N$$ (12)

$$\left\{\begin{array}{l} a=\min\;\left(x_{i},y_{i}\right),\\ b=\max\;\left(x_{i},y_{i}\right)-\min\;\left(x_{i},y_{i}\right), \end{array}\right.\begin{array}{cc} & i=0,1,2, \end{array}\ldots ,N$$ (13)

**FIGURE 3:**
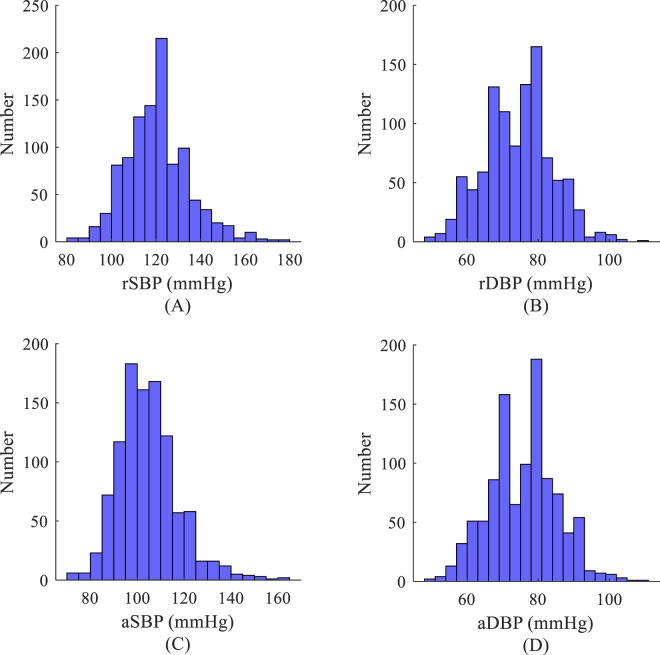
Histogram of blood pressure distribution based on the measurement data set. **(A)** Systolic blood pressure of the radial artery; **(B)** Diastolic blood pressure of the radial artery; **(C)** Systolic blood pressure of the aorta; **(D)** Diastolic blood pressure of the aorta.

Here $x_{i}$ , $y_{i}$ denote the input radial artery pressure waveform and the actual central artery pressure waveform signals, respectively. ${\tilde{x}}_{i}$ and ${\tilde{y}}_{i}$ represent the normalized radial artery pressure waveform signal and the actual central artery pressure waveform signal, respectively. As shown in Figure 3, for the measurement dataset (NEU-PWDB), the mean value of diastolic blood pressure at the radial artery is 49 mmHg; the mean value of systolic blood pressure is 178 mmHg. Considering the combined dataset, Equations 12, 13, a takes the value of 45 mmHg and b takes the value of 135 mmHg.

## 4 Comparative analysis of experimental results

### 4.1 Evaluation indices

#### 4.1.1 Mean absolute error

Mean absolute error (MAE) is a commonly used evaluation index. It calculates the average of the absolute value of the prediction error of each sample, reflecting the average error magnitude. The advantage of MAE is that it intuitively demonstrates the magnitude of the gap between the model’s predicted values and the actual values, which does not consider the positive or negative of the expected value. Also, MAE pays more attention to the size of the absolute error, which makes it more reflective of the degree of deviation of the predicted value than the mean squared error. The range of values of the MAE is [0, + ∞ ), and it will be more accurate when the predicted value and the real value match exactly is equal to 0, i.e., the perfect model; the larger the error, the larger the value. In Equation 14, N is the number of samples, $y_{i}$ is the actual value of the first i sample, $\hat{y_{i}}$ is the predicted value of the first i sample.

$$MAE=\frac{1}{N}\sum_{i=1}^{N}\left|y_{i}-\hat{y_{i}}\right|$$ (14)

#### 4.1.2 Root mean square error

Root mean square error (RMSE) is a normal indicator to assess prediction models’ accuracy. It measures the degree of deviation between the predicted and the actual value. It is calculated as the square root of the sum of the squares of the difference between the predicted and the actual value. The value of RMSE ranges from [0, + ∞ ). The smaller the RMSE is, the smaller the prediction error of the model is, and the better the prediction ability of the model. The specific formula is as follows in Equation 15:

$$RMSE=\sqrt{\frac{1}{N}\sum_{i=1}^{N}{\left(y_{i}-\hat{y_{i}}\right)}^{2}\;}$$ (15)

#### 4.1.3 Maximum error

Maximum error (MAX error) is one indicator for assessing the model’s prediction performance, indicating the maximum value of the absolute error between the predicted and the actual value in all samples. Its core focus is on the model’s performance in the worst case, rather than the average or overall error level. The specific formulas are given as in Equation 8:

$$\mathrm{MAX}\;Error=\max\left(\left|y_{1}-{\hat{y}}_{1}\right|,\ldots ,\left|y_{N}-{\hat{y}}_{N}\right|\right)$$ (16)

#### 4.1.4 Spearman’s correlation coefficient

Spearman’s correlation coefficient (SCC) is a non-parametric statistic that measures the monotonic relationship between two variables. It is based on the rank order of the data rather than the raw values. Therefore, it does not require the data to satisfy the assumptions of a normal distribution or a linear relationship. This also leads to its difference from the Pearson correlation coefficient, which is less sensitive to outliers. At the same time, SCC is effective in capturing monotonic relationships between variables, whether linear or nonlinear. Where N is the number of samples, $d_{i}$ is the rank difference of the first i sample of the two variables.i.e., the difference between the true value’s rank and the predicted value’s rank. The specific formula is as follows in Equation 17:

$$SCC=1-\frac{6\sum_{i=1}^{N}{d_{i}}^{2}}{N\left(N^{2}-1\right)}$$ (17)

### 4.2 Experimental results

**FIGURE 4:**
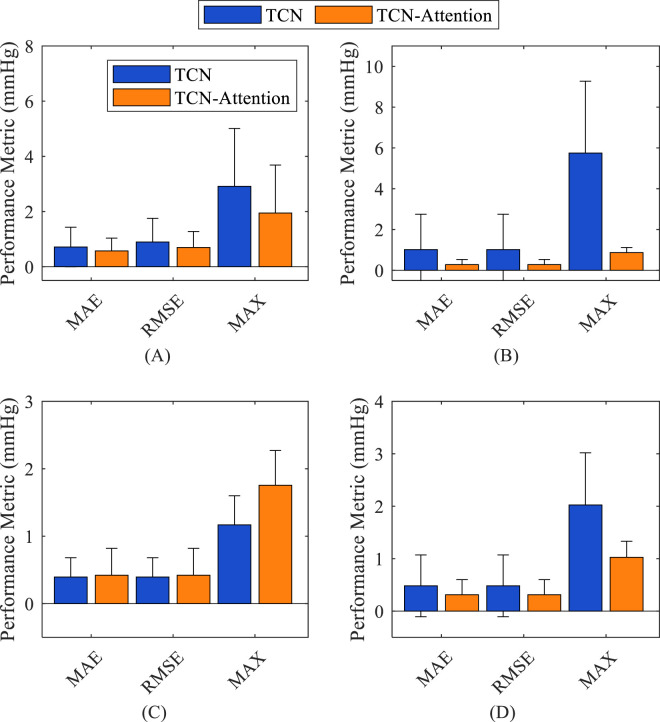
Comparison of the accuracy of the prediction results of different models on different indices. **(A)** total waveform (TW); **(B)** systolic blood pressure (SBP); **(C)** diastolic blood pressure (DBP); **(D)** mean arterial pressure (MAP).

As shown in Figure 4, the TCN-Attention-based model is superior to the TCN model in reconstructing central aortic pressure. The prediction errors of the TCN-Attention model were significantly lower than those of the TCN model (p < 0.05) in terms of the MAE, RMSE, MAX error assessment indices, as well as the total waveform, systolic blood pressure, and mean arterial pressure, which verified that the attention mechanism enhances temporal feature extraction. For diastolic blood pressure prediction, the performance of the two models was comparable, and the TCN-Attention-based model was slightly better regarding the result indicators. As shown in Table 2, the result of TCN-Attention model was significantly lower in reconstructing the entire waveform of central aortic pressure (MAE: 0.57 ± 0.46 mmHg) than the TCN model (MAE: 0.71 ± 0.71 mmHg); the result of TCN-Attention model (RMSE: 0.69 ± 0.57 mmHg) was significantly lower than the TCN model (RMSE: 0.89 ± 0.86 mmHg); the MAX value based on the TCN-Attention model (MAX: 1.94 ± 1.73 mmHg) was significantly lower than that of the TCN model (MAX: 2.91 ± 2.09 mmHg). The TCN-Attention model outperforms the TCN model in the overall waveform prediction, and in particular, the performance of TCN-Attention model is outstanding in the control of the maximum error, with an error reduction of 33%. The results of TCN-Attention model were significantly lower than the TCN model in estimating systolic blood pressure (MAE and RMSE: 0.27 ± 0.25 mmHg) (MAE and RMSE: 1.01 ± 1.73 mmHg). The result of TCN-Attention model was significantly lower in estimating systolic blood pressure (MAX: 0.86 ± 0.24 mmHg) than the TCN model (MAX: 5.74 ± 3.51 mmHg). The TCN-Attention model performed very well in SBP prediction, with an error reduction of more than 70%, and the maximum error reduction was 85%. The results of TCN-Attention model were slightly higher than the TCN model in estimating diastolic blood pressure (MAE and RMSE: 0.42 ± 0.39 mmHg), and the result of the TCN-Attention model (Max Error: 1.75 ± 0.51 mmHg) was slightly higher than the TCN model (Max Error: 1.16 ± 0.43 mmHg). However, the difference was insignificant.

**TABLE 2:** Comparison of the accuracy of prediction results of different models on different indices (Mean ± standard deviation in mmHg).

Methods	BP	MAE	RMSE	Max error
TCN	TW	0.71 ± 0.71	0.89 ± 0.86	2.91 ± 2.09
	SBP	1.01 ± 1.73	1.01 ± 1.73	5.74 ± 3.51
	DBP	0.39 ± 0.28	0.39 ± 0.28	1.16 ± 0.43
	MAP	0.48 ± 0.58	0.48 ± 0.58	2.02 ± 1.02
TCN-Attention	TW	0.57 ± 0.46*	0.69 ± 0.57	1.94 ± 1.73*
	SBP	0.27 ± 0.25**	0.27 ± 0.25**	0.86 ± 0.24**
	DBP	0.42 ± 0.39	0.42 ± 0.39	1.75 ± 0.51
	MAP	0.31 ± 0.28*	0.31 ± 0.28*	0.99 ± 0.30*

^*^indicates P < 0.05;^ **^ indicates P < 0.01.

**FIGURE 5:**
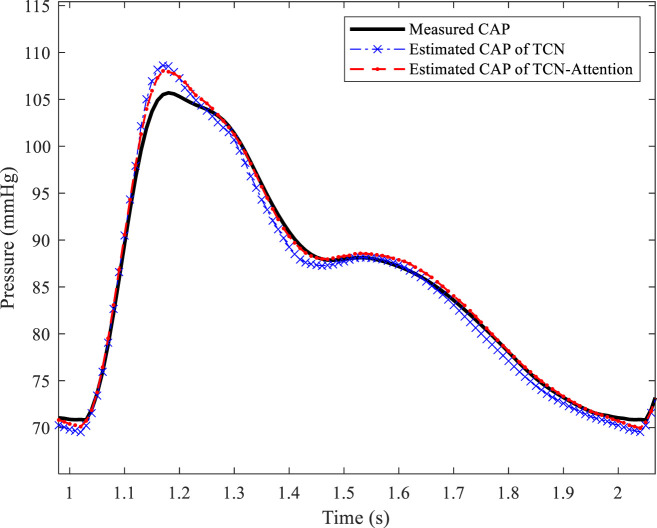
Estimated central aortic pressure waveform based on different models.

The result of the TCN model was slightly better than the TCN-Attention model in DBP prediction, but did not reach statistical significance, which the physiological fluctuation of DBP is relatively light. The results of the TCN-Attention model were significantly lower than the TCN model (MAE and RMSE: 0.31 ± 0.28 mmHg) in estimating mean arterial pressure (MAP) (MAE and RMSE: 0.48 ± 0.58 mmHg); the TCN-Attention model (MAX: 0.99 ± 0.30 mmHg) was significantly lower than the TCN model (MAX: 2.02 ± 1.02 mmHg). The TCN-Attention model significantly outperformed the TCN in MAP prediction, with more than 35% reduction in error and 51% reduction in maximum deviation, improving stability significantly. The SCC of the total waveform for both models was as high as 0.9985 and 0.9970, indicating that both could capture the trend of blood pressure changes well. As shown in Figure 5, the central aortic pressure waveform estimated by the two different methods, the blood pressure estimation based on the TCN-Attention model, are closer to the real measured central aortic pressure waveform than the TCN model in the total waveform and the key feature points.

## 5 Discussion and conclusion

The TCN-Attention model outperforms the traditional TCN model in most metrics, especially in systolic blood pressure estimation. It indicates that the attention mechanism effectively captures the key features in the blood pressure signals. DBP has limited room for model improvement due to small physiological fluctuations. TCN-Attention is a deep learning model that combines time-series convolution networks and the attention mechanism architecture designed for time series prediction tasks. The model utilizes temporal convolution layers to capture local and long-term dependencies of time series data. The attention mechanism realizes dynamic allocation of weights at different time steps to highlight essential features. The standard deviations of TCN-Attention metrics are generally smaller than those of TCN, indicating that its prediction results are more stable and reliable. The SCCs of both models are as high as 0.9985 and 0.9970, suggesting that both can capture the blood pressure trend well.

The total waveform usually contains full-cycle features of the blood pressure signal, such as systole, diastole, and pulse wave morphology, which have a wide range of numerical variations and high dynamics. The model prediction needs to capture various features simultaneously, leading to different MAE and RMSE sensitivities to other types of errors (e.g., systematic deviation and random fluctuation). The distribution of errors across the waveform metrics may exhibit a long-tailed distribution, with a few samples containing substantial errors. The RMSE values are sensitive to outliers, while the MAE is more robust.

The estimation of systolic, diastolic, and mean arterial pressure relies on identifying specific critical points in a continuous physiological signal. When a model’s prediction error stems predominantly from systematic bias—such as a consistent overestimation or underestimation across measurements—the error distribution for each of these point estimates may approximate a fixed-offset distribution. In such cases, the MAE and RMSE values tend to converge, as the uniform nature of the systematic offset diminishes the influence of outlier-dependent penalization in RMSE. Considering individual variations and the specific prediction requirements for special populations (e.g., hypertensive patients), multimodal data fusion has been actively explored, for instance, by integrating photoplethysmography signals with complementary physiological data. Future efforts could incorporate a wider array of physiological features, such as heart rate variability, to further enhance the accuracy and robustness of predictive models.

## Data Availability

The original contributions presented in the study are included in the article/supplementary material, further inquiries can be directed to the corresponding authors.

## References

[B1] Bai S. Kolter J. Z. Koltun V. (2018). An empirical evaluation of generic convolutional and recurrent networks for sequence modeling. arXiv preprint arXiv:1803.01271.

[B2] Cameron J. D. McGrath B. P. Dart A. M. (1998). Use of radial artery applanation tonometry and a generalized transfer function to determine aortic pressure augmentation in subjects with treated hypertension. J. Am. Coll. Cardiol. 32 (5), 1214–1220. 10.1016/s0735-1097(98)00411-2 9809928

[B4] Chen Y. Zhang Y. Jiang M. Li J. Han X. Sun K. (2025). SFAG-DeepLabv3+: an automatic segmentation approach for coronary angiography images. Neurocomputing 650, 130781. 10.1016/j.neucom.2025.130781

[B5] Ding F. H. Fan W. X. Zhang R. Y. Zhang Q. Li Y. Wang J. G. (2011). Validation of the noninvasive assessment of central blood pressure by the SphygmoCor and Omron devices against the invasive catheter measurement. Am. J. Hypertens. 24 (12), 1306–1311. 10.1038/ajh.2011.145 21976274

[B6] Du S. Liu W. Yao Y. Sun G. He Y. Alastruey J. (2022). Reconstruction of the aortic pressure waveform using a two-level adaptive transfer function strategy. Measurement 204 (30), 112111. 10.1016/j.measurement.2022.112111

[B7] Gu J. Wang Z. Kuen J. Ma L. Shahroudy A. Shuai B. (2018). Recent advances in convolutional neural networks. Pattern Recognit. 77, 354–377. 10.1016/j.patcog.2017.10.013

[B8] He K. Zhang X. Ren S. Sun J. (2016). “Deep residual learning for image recognition [C],” in Proceedings of the IEEE Conference on Computer Vision and Pattern Recognition, 770–778.

[B10] Kroeker E. J. Wood E. H. (1955). Comparison of simultaneously recorded central and peripheral arterial pressure pulses during rest, exercise and tilted position in man. Circulation Res. 3 (6), 623–632. 10.1161/01.res.3.6.623 13270378

[B11] Lin Y. Koprinska I. Rana M. (2021). “Temporal convolutional attention neural networks for time series forecasting [C],” in 2021 International Joint Conference on Neural Networks (IJCNN) (IEEE), 1–8.

[B12] McGaughey T. J. Fletcher E. A. Shah S. A. (2016). Impact of antihypertensive agents on central systolic blood pressure and augmentation index: a meta-analysis. Am. J. Hypertens. 29 (4), 448–457. 10.1093/ajh/hpv134 26289583 PMC4886490

[B13] Morgan T. Lauri J. Bertram D. Anderson A. (2004). Effect of different antihypertensive drug classes on central aortic pressure. Am. J. Hypertens. 17 (2), 118–123. 10.1016/j.amjhyper.2003.09.012 14751652

[B14] Ohte N. Saeki T. Miyabe H. Sakata S. Mukai S. Hayano J. (2007). Relationship between blood pressure obtained from the upper arm with a cuff-type sphygmomanometer and central blood pressure measured with a catheter-tipped micromanometer. Heart Vessels 22 (6), 410–415. 10.1007/s00380-007-0998-5 18044000

[B15] Payne R. A. Teh C. H. Webb D. J. Maxwell S. R. J. (2007). A generalized arterial transfer function derived at rest underestimates augmentation of central pressure after exercise. J. Hypertens. 25 (11), 2266–2272. 10.1097/HJH.0b013e3282ef96fa 17921821

[B16] Roman M. J. Devereux R. B. Kizer J. R. Lee E. T. Galloway J. M. Ali T. (2007). Central pressure more strongly relates to vascular disease and outcome than does brachial pressure: the strong heart study. Hypertension 50 (1), 197–203. 10.1161/HYPERTENSIONAHA.107.089078 17485598

[B18] Szegedy C. Liu W. Jia Y. Sermanet P. Reed S. Anguelov D. (2015). “Going deeper with convolutions [C],” in Proceedings of the IEEE conference on computer vision and pattern recognition, 1–9.

[B19] Vos T. Lim S. S. Abbafati C. Abbas K. M. Abbasi M. Abbasifard M. (2020). Global burden of 369 diseases and injuries in 204 countries and territories, 1990–2019: a systematic analysis for the Global Burden of Disease Study 2019. Lancet 396 (10258), 1204–1222. 10.1016/S0140-6736(20)30925-9 33069326 PMC7567026

[B20] Wang T. Wu D. Coates A. andNg A. (2012). End-to-end text recognition with convolutional neural networks [C] International Conference on Pattern Recognition (ICPR), 3304–3308.

[B21] Wang S. Li Y. Liu Z. Liu X. (2025). Intelligent faulty diagnosis on photovoltaic farm based on spatial attention mechanism and temporal convolutional network. Next Res. 2 (3), 100604. 10.1016/j.nexres.2025.100604

[B22] World Health Organization (2018). Noncommunicable diseases country profiles 2018 [R]. Geneva: World Health Organization.

[B23] Zeiler M. D. Fergus R. (2014). “Visualizing and understanding convolutional networks [C],” in European conference on computer vision (Cham: Springer), 818–833.

[B24] Zhou S. Xu K. Fang Y. Alastruey J. Vennin S. Yang J. (2024). Patient-specific non-invasive estimation of the aortic blood pressure waveform by Ultrasound and Tonometry. Comput. Methods Programs Biomed. 247, 108082. 10.1016/j.cmpb.2024.108082 38422893

[B25] Zuo J. L. Li Y. Yan Z. J. Zhang R. Y. Shen W. F. Zhu D. L. (2010). Validation of the central blood pressure estimation by the SphygmoCor system in Chinese. Blood Press. Monit. 15 (5), 268–274. 10.1097/MBP.0b013e3283386866 20357649

